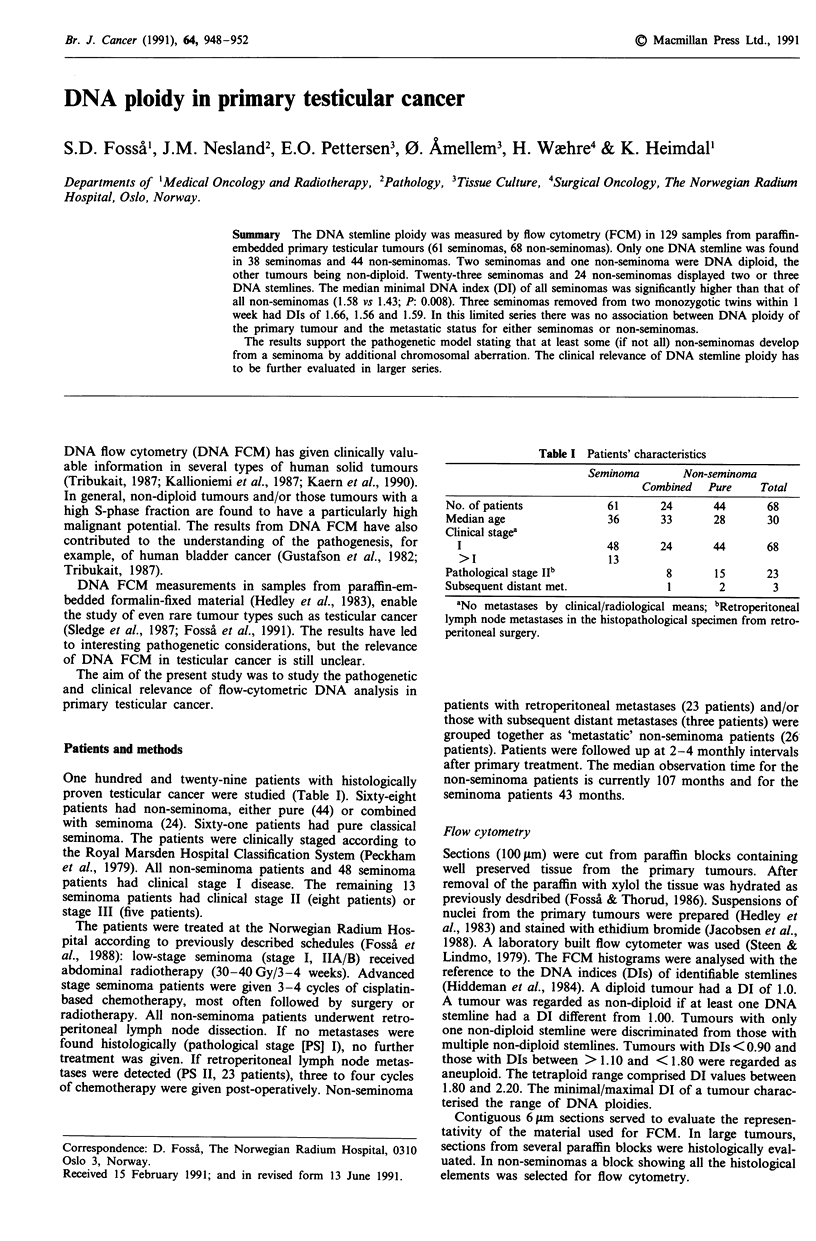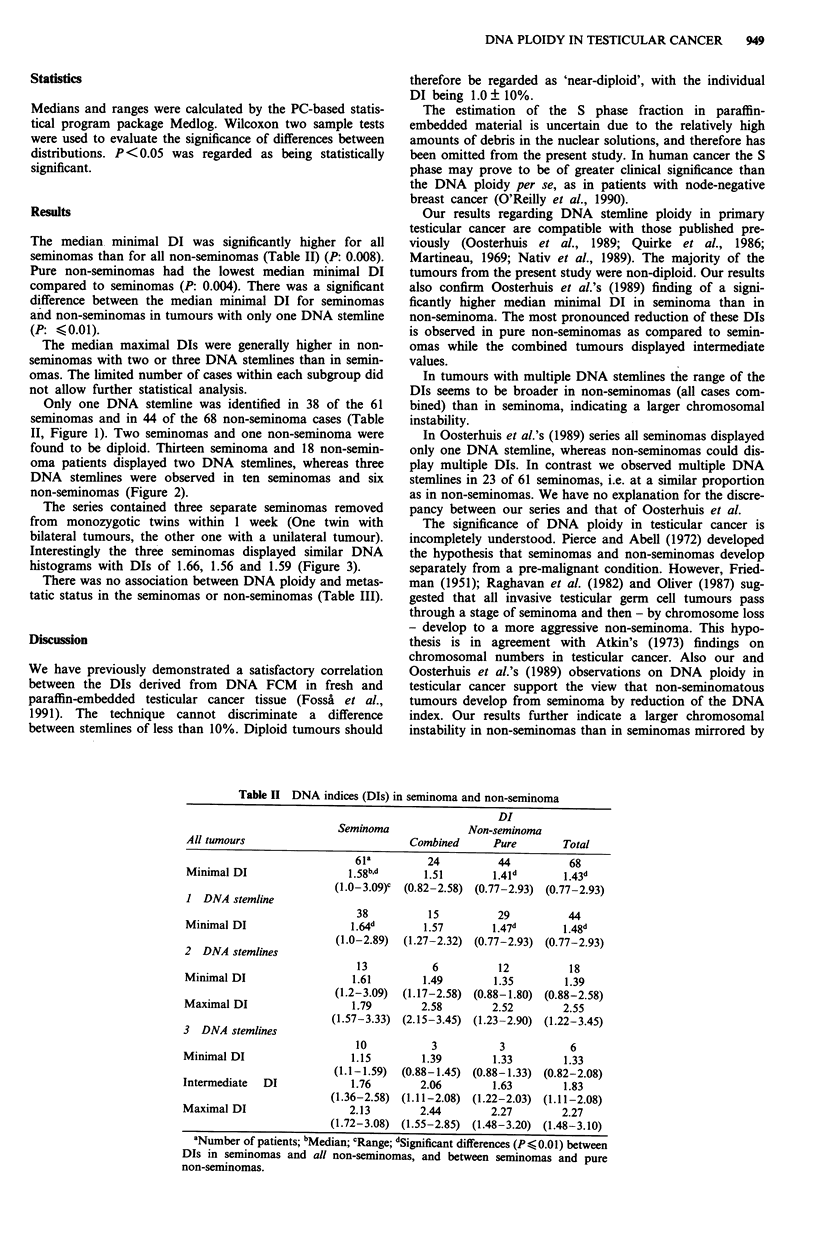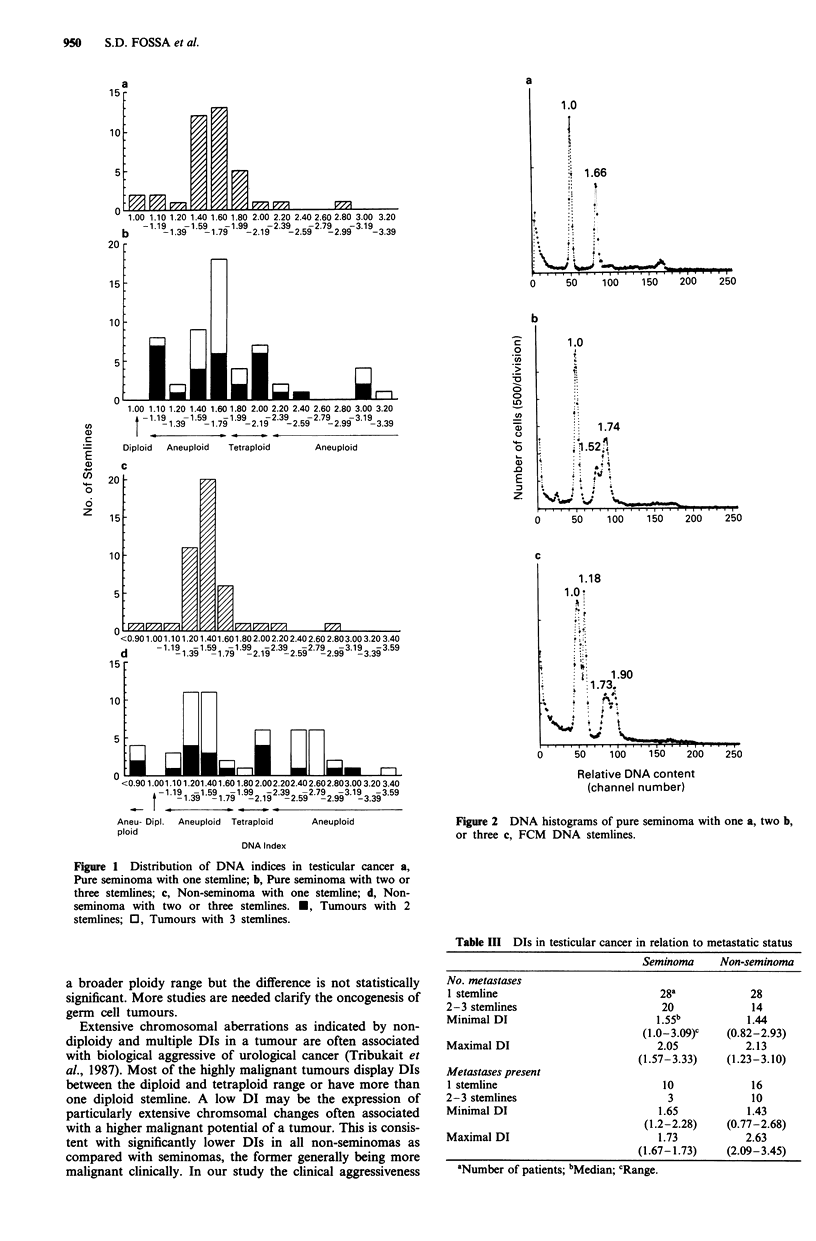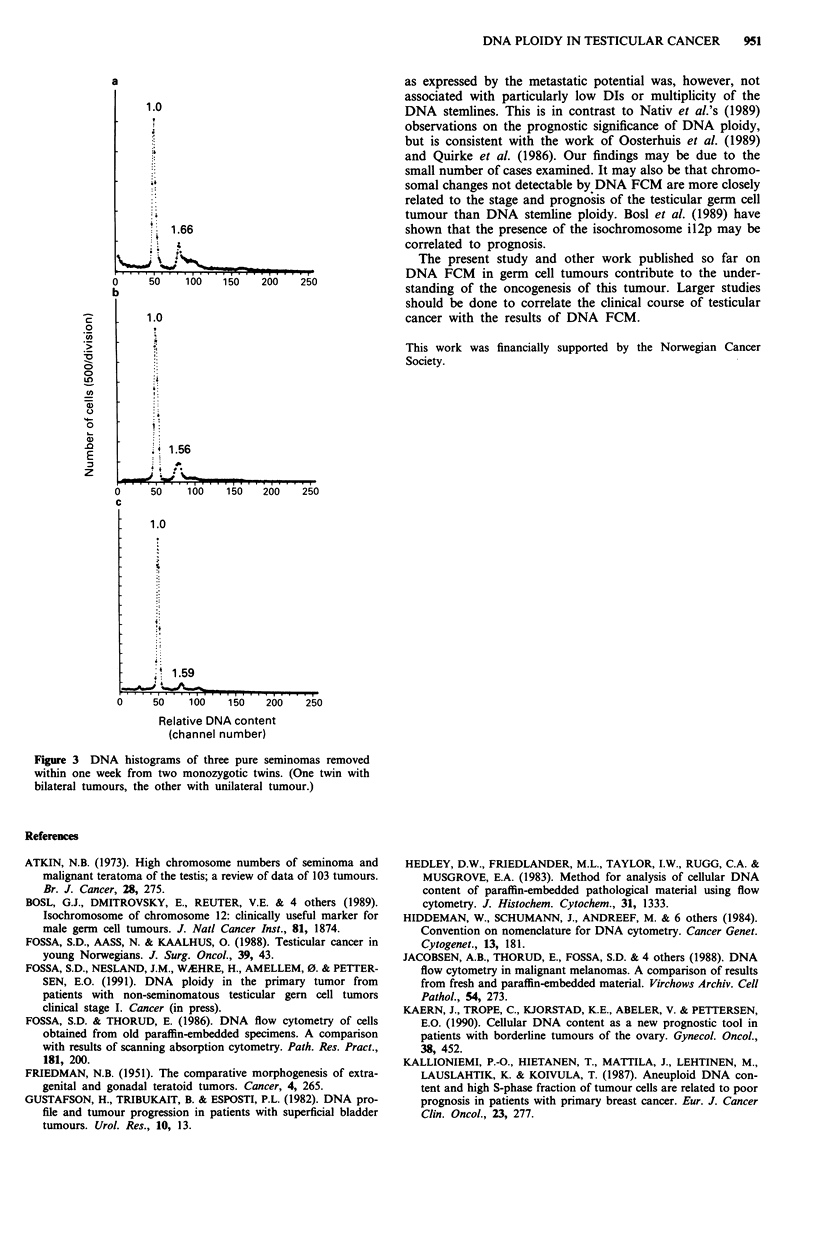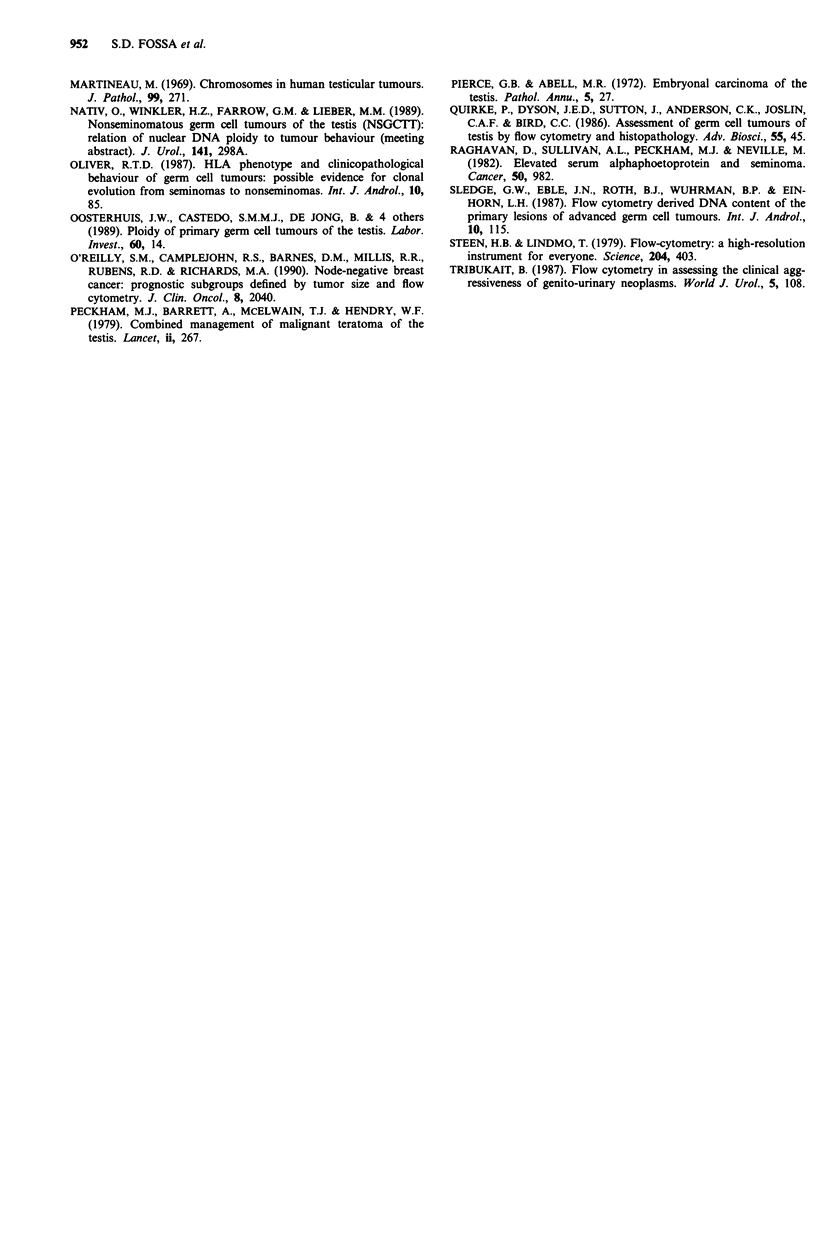# DNA ploidy in primary testicular cancer.

**DOI:** 10.1038/bjc.1991.432

**Published:** 1991-11

**Authors:** S. D. Fosså, J. M. Nesland, E. O. Pettersen, O. Amellem, H. Waehre, K. Heimdal

**Affiliations:** Department of Medical Oncology and Radiotherapy, Norwegian Radium Hospital, Oslo.

## Abstract

The DNA stemline ploidy was measured by flow cytometry (FCM) in 129 samples from paraffin-embedded primary testicular tumours (61 seminomas, 68 non-seminomas). Only one DNA stemline was found in 38 seminomas and 44 non-seminomas. Two seminomas and one non-seminoma were DNA diploid, the other tumours being non-diploid. Twenty-three seminomas and 24 non-seminomas displayed two or three DNA stemlines. The median minimal DNA index (DI) of all seminomas was significantly higher than that of all non-seminomas (1.58 vs 1.43; P: 0.008). Three seminomas removed from two monozygotic twins within 1 week had DIs of 1.66, 1.56 and 1.59. In this limited series there was no association between DNA ploidy of the primary tumour and the metastatic status for either seminomas or non-seminomas. The results support the pathogenetic model stating that at least some (if not all) non-seminomas develop from a seminoma by additional chromosomal aberration. The clinical relevance of DNA stemline ploidy has to be further evaluated in larger series.


Br. J. Cancer (1991), 64, 948–952 © Macmillan Press Ltd., 1991

### DNA ploidy in primary testicular cancer

S.D. Fosså[1], J.M. Nesland[2], E.O. Pettersen[3], Ø. Åmellem[3], H. Wæhre[4] & K. Heimdal[1]

*Departments of [1]Medical Oncology and Radiotherapy, [2]Pathology, [3]Tissue Culture, [4]Surgical Oncology, The Norwegian Radium Hospital, Oslo, Norway.*

**Summary** The DNA stemline ploidy was measured by flow cytometry (FCM) in 129 samples from paraffin-embedded primary testicular tumours (61 seminomas, 68 non-seminomas). Only one DNA stemline was found in 38 seminomas and 44 non-seminomas. Two seminomas and one non-seminoma were DNA diploid, the other tumours being non-diploid. Twenty-three seminomas and 24 non-seminomas displayed two or three DNA stemlines. The median minimal DNA index (DI) of all seminomas was significantly higher than that of all non-seminomas (1.58 *vs* 1.43; *P*: 0.008). Three seminomas removed from two monozygotic twins within 1 week had DIs of 1.66, 1.56 and 1.59. In this limited series there was no association between DNA ploidy of the primary tumour and the metastatic status for either seminomas or non-seminomas.

The results support the pathogenetic model stating that at least some (if not all) non-seminomas develop from a seminoma by additional chromosomal aberration. The clinical relevance of DNA stemline ploidy has to be further evaluated in larger series.

DNA flow cytometry (DNA FCM) has given clinically valuable information in several types of human solid tumours (Tribukait, 1987; Kallioniemi *et al.*, 1987; Kaern *et al.*, 1990). In general, non-diploid tumours and/or those tumours with a high S-phase fraction are found to have a particularly high malignant potential. The results from DNA FCM have also contributed to the understanding of the pathogenesis, for example, of human bladder cancer (Gustafson *et al.*, 1982; Tribukait, 1987).

DNA FCM measurements in samples from paraffin-embedded formalin-fixed material (Hedley *et al.*, 1983), enable the study of even rare tumour types such as testicular cancer (Sledge *et al.*, 1987; Fosså *et al.*, 1991). The results have led to interesting pathogenetic considerations, but the relevance of DNA FCM in testicular cancer is still unclear.

The aim of the present study was to study the pathogenetic and clinical relevance of flow-cytometric DNA analysis in primary testicular cancer.

### Patients and methods

One hundred and twenty-nine patients with histologically proven testicular cancer were studied (Table I). Sixty-eight patients had non-seminoma, either pure (44) or combined with seminoma (24). Sixty-one patients had pure classical seminoma. The patients were clinically staged according to the Royal Marsden Hospital Classification System (Peckham *et al.*, 1979). All non-seminoma patients and 48 seminoma patients had clinical stage I disease. The remaining 13 seminoma patients had clinical stage II (eight patients) or stage III (five patients).

The patients were treated at the Norwegian Radium Hospital according to previously described schedules (Fosså *et al.*, 1988): low-stage seminoma (stage I, IIA/B) received abdominal radiotherapy (30–40 Gy/3–4 weeks). Advanced stage seminoma patients were given 3–4 cycles of cisplatin-based chemotherapy, most often followed by surgery or radiotherapy. All non-seminoma patients underwent retroperitoneal lymph node dissection. If no metastases were found histologically (pathological stage [PS] I), no further treatment was given. If retroperitoneal lymph node metastases were detected (PS II, 23 patients), three to four cycles of chemotherapy were given post-operatively. Non-seminoma patients with retroperitoneal metastases (23 patients) and/or those with subsequent distant metastases (three patients) were grouped together as 'metastatic' non-seminoma patients (26 patients). Patients were followed up at 2–4 monthly intervals after primary treatment. The median observation time for the non-seminoma patients is currently 107 months and for the seminoma patients 43 months.

**Table I** Patients' characteristics

| | *Seminoma* | *Non-seminoma* | | |
|---|---|---|---|---|
| | | *Combined* | *Pure* | *Total* |
| No. of patients | 61 | 24 | 44 | 68 |
| Median age | 36 | 33 | 28 | 30 |
| Clinical stage[a] | | | | |
| I | 48 | 24 | 44 | 68 |
| >I | 13 | | | |
| Pathological stage II[b] | | 8 | 15 | 23 |
| Subsequent distant met. | | 1 | 2 | 3 |

[a]No metastases by clinical/radiological means; [b]Retroperitoneal lymph node metastases in the histopathological specimen from retroperitoneal surgery.

### *Flow cytometry*

Sections (100 µm) were cut from paraffin blocks containing well preserved tissue from the primary tumours. After removal of the paraffin with xylol the tissue was hydrated as previously desdribed (Fosså & Thorud, 1986). Suspensions of nuclei from the primary tumours were prepared (Hedley *et al.*, 1983) and stained with ethidium bromide (Jacobsen *et al.*, 1988). A laboratory built flow cytometer was used (Steen & Lindmo, 1979). The FCM histograms were analysed with the reference to the DNA indices (DIs) of identifiable stemlines (Hiddeman *et al.*, 1984). A diploid tumour had a DI of 1.0. A tumour was regarded as non-diploid if at least one DNA stemline had a DI different from 1.00. Tumours with only one non-diploid stemline were discriminated from those with multiple non-diploid stemlines. Tumours with DIs <0.90 and those with DIs between >1.10 and <1.80 were regarded as aneuploid. The tetraploid range comprised DI values between 1.80 and 2.20. The minimal/maximal DI of a tumour characterised the range of DNA ploidies.

Contiguous 6 µm sections served to evaluate the representativity of the material used for FCM. In large tumours, sections from several paraffin blocks were histologically evaluated. In non-seminomas a block showing all the histological elements was selected for flow cytometry.

Correspondence: D. Fosså, The Norwegian Radium Hospital, 0310 Oslo 3, Norway.
Received 15 February 1991; and in revised form 13 June 1991.

### Statistics

Medians and ranges were calculated by the PC-based statistical program package Medlog. Wilcoxon two sample tests were used to evaluate the significance of differences between distributions. $P<0.05$ was regarded as being statistically significant.

### Results

The median minimal DI was significantly higher for all seminomas than for all non-seminomas (Table II) (*P*: 0.008). Pure non-seminomas had the lowest median minimal DI compared to seminomas (*P*: 0.004). There was a significant difference between the median minimal DI for seminomas and non-seminomas in tumours with only one DNA stemline (*P*: $\leqslant 0.01$).

The median maximal DIs were generally higher in non-seminomas with two or three DNA stemlines than in seminomas. The limited number of cases within each subgroup did not allow further statistical analysis.

Only one DNA stemline was identified in 38 of the 61 seminomas and in 44 of the 68 non-seminoma cases (Table II, Figure 1). Two seminomas and one non-seminoma were found to be diploid. Thirteen seminoma and 18 non-seminoma patients displayed two DNA stemlines, whereas three DNA stemlines were observed in ten seminomas and six non-seminomas (Figure 2).

The series contained three separate seminomas removed from monozygotic twins within 1 week (One twin with bilateral tumours, the other one with a unilateral tumour). Interestingly the three seminomas displayed similar DNA histograms with DIs of 1.66, 1.56 and 1.59 (Figure 3).

There was no association between DNA ploidy and metastatic status in the seminomas or non-seminomas (Table III).

### Discussion

We have previously demonstrated a satisfactory correlation between the DIs derived from DNA FCM in fresh and paraffin-embedded testicular cancer tissue (Fosså *et al*., 1991). The technique cannot discriminate a difference between stemlines of less than 10%. Diploid tumours should therefore be regarded as 'near-diploid', with the individual DI being $1.0 \pm 10\%$.

The estimation of the S phase fraction in paraffin-embedded material is uncertain due to the relatively high amounts of debris in the nuclear solutions, and therefore has been omitted from the present study. In human cancer the S phase may prove to be of greater clinical significance than the DNA ploidy *per se*, as in patients with node-negative breast cancer (O'Reilly *et al*., 1990).

Our results regarding DNA stemline ploidy in primary testicular cancer are compatible with those published previously (Oosterhuis *et al*., 1989; Quirke *et al*., 1986; Martineau, 1969; Nativ *et al*., 1989). The majority of the tumours from the present study were non-diploid. Our results also confirm Oosterhuis *et al*.'s (1989) finding of a significantly higher median minimal DI in seminoma than in non-seminoma. The most pronounced reduction of these DIs is observed in pure non-seminomas as compared to seminomas while the combined tumours displayed intermediate values.

In tumours with multiple DNA stemlines the range of the DIs seems to be broader in non-seminomas (all cases combined) than in seminoma, indicating a larger chromosomal instability.

In Oosterhuis *et al*.'s (1989) series all seminomas displayed only one DNA stemline, whereas non-seminomas could display multiple DIs. In contrast we observed multiple DNA stemlines in 23 of 61 seminomas, i.e. at a similar proportion as in non-seminomas. We have no explanation for the discrepancy between our series and that of Oosterhuis *et al.*

The significance of DNA ploidy in testicular cancer is incompletely understood. Pierce and Abell (1972) developed the hypothesis that seminomas and non-seminomas develop separately from a pre-malignant condition. However, Friedman (1951); Raghavan *et al*. (1982) and Oliver (1987) suggested that all invasive testicular germ cell tumours pass through a stage of seminoma and then – by chromosome loss – develop to a more aggressive non-seminoma. This hypothesis is in agreement with Atkin's (1973) findings on chromosomal numbers in testicular cancer. Also our and Oosterhuis *et al*.'s (1989) observations on DNA ploidy in testicular cancer support the view that non-seminomatous tumours develop from seminoma by reduction of the DNA index. Our results further indicate a larger chromosomal instability in non-seminomas than in seminomas mirrored by

**Table II** DNA indices (DIs) in seminoma and non-seminoma

| | | *DI* | | |
|---|---|---|---|---|
| | *Seminoma* | | *Non-seminoma* | |
| *All tumours* | | *Combined* | *Pure* | *Total* |
| | 61[a] | 24 | 44 | 68 |
| Minimal DI | 1.58[b,d] | 1.51 | 1.41[d] | 1.43[d] |
| | (1.0–3.09)[c] | (0.82–2.58) | (0.77–2.93) | (0.77–2.93) |
| *1 DNA stemline* | | | | |
| | 38 | 15 | 29 | 44 |
| Minimal DI | 1.64[d] | 1.57 | 1.47[d] | 1.48[d] |
| | (1.0–2.89) | (1.27–2.32) | (0.77–2.93) | (0.77–2.93) |
| *2 DNA stemlines* | | | | |
| | 13 | 6 | 12 | 18 |
| Minimal DI | 1.61 | 1.49 | 1.35 | 1.39 |
| | (1.2–3.09) | (1.17–2.58) | (0.88–1.80) | (0.88–2.58) |
| Maximal DI | 1.79 | 2.58 | 2.52 | 2.55 |
| | (1.57–3.33) | (2.15–3.45) | (1.23–2.90) | (1.22–3.45) |
| *3 DNA stemlines* | | | | |
| | 10 | 3 | 3 | 6 |
| Minimal DI | 1.15 | 1.39 | 1.33 | 1.33 |
| | (1.1–1.59) | (0.88–1.45) | (0.88–1.33) | (0.82–2.08) |
| Intermediate DI | 1.76 | 2.06 | 1.63 | 1.83 |
| | (1.36–2.58) | (1.11–2.08) | (1.22–2.03) | (1.11–2.08) |
| Maximal DI | 2.13 | 2.44 | 2.27 | 2.27 |
| | (1.72–3.08) | (1.55–2.85) | (1.48–3.20) | (1.48–3.10) |

[a]Number of patients; [b]Median; [c]Range; [d]Significant differences ($P \leqslant 0.01$) between DIs in seminomas and *all* non-seminomas, and between seminomas and pure non-seminomas.

**Figure 1** Distribution of DNA indices in testicular cancer **a**, Pure seminoma with one stemline; **b**, Pure seminoma with two or three stemlines; **c**, Non-seminoma with one stemline; **d**, Non-seminoma with two or three stemlines. ■, Tumours with 2 stemlines; □, Tumours with 3 stemlines.

**Figure 2** DNA histograms of pure seminoma with one **a**, two **b**, or three **c**, FCM DNA stemlines.

a broader ploidy range but the difference is not statistically significant. More studies are needed clarify the oncogenesis of germ cell tumours.

Extensive chromosomal aberrations as indicated by non-diploidy and multiple DIs in a tumour are often associated with biological aggressive of urological cancer (Tribukait *et al.*, 1987). Most of the highly malignant tumours display DIs between the diploid and tetraploid range or have more than one diploid stemline. A low DI may be the expression of particularly extensive chromsomal changes often associated with a higher malignant potential of a tumour. This is consistent with significantly lower DIs in all non-seminomas as compared with seminomas, the former generally being more malignant clinically. In our study the clinical aggressiveness

**Table III** DIs in testicular cancer in relation to metastatic status

| | *Seminoma* | *Non-seminoma* |
|---|---|---|
| *No. metastases* | | |
| 1 stemline | 28[a] | 28 |
| 2–3 stemlines | 20 | 14 |
| Minimal DI | 1.55[b] | 1.44 |
| | (1.0–3.09)[c] | (0.82–2.93) |
| Maximal DI | 2.05 | 2.13 |
| | (1.57–3.33) | (1.23–3.10) |
| *Metastases present* | | |
| 1 stemline | 10 | 16 |
| 2–3 stemlines | 3 | 10 |
| Minimal DI | 1.65 | 1.43 |
| | (1.2–2.28) | (0.77–2.68) |
| Maximal DI | 1.73 | 2.63 |
| | (1.67–1.73) | (2.09–3.45) |

[a]Number of patients; [b]Median; [c]Range.

**Figure 3** DNA histograms of three pure seminomas removed within one week from two monozygotic twins. (One twin with bilateral tumours, the other with unilateral tumour.)

as expressed by the metastatic potential was, however, not associated with particularly low DIs or multiplicity of the DNA stemlines. This is in contrast to Nativ *et al.*'s (1989) observations on the prognostic significance of DNA ploidy, but is consistent with the work of Oosterhuis *et al.* (1989) and Quirke *et al.* (1986). Our findings may be due to the small number of cases examined. It may also be that chromosomal changes not detectable by DNA FCM are more closely related to the stage and prognosis of the testicular germ cell tumour than DNA stemline ploidy. Bosl *et al.* (1989) have shown that the presence of the isochromosome i12p may be correlated to prognosis.

The present study and other work published so far on DNA FCM in germ cell tumours contribute to the understanding of the oncogenesis of this tumour. Larger studies should be done to correlate the clinical course of testicular cancer with the results of DNA FCM.

This work was financially supported by the Norwegian Cancer Society.

### References

ATKIN, N.B. (1973). High chromosome numbers of seminoma and malignant teratoma of the testis; a review of data of 103 tumours. *Br. J. Cancer*, **28**, 275.

BOSL, G.J., DMITROVSKY, E., REUTER, V.E. & 4 others (1989). Isochromosome of chromosome 12: clinically useful marker for male germ cell tumours. *J. Natl Cancer Inst.*, **81**, 1874.

FOSSA, S.D., AASS, N. & KAALHUS, O. (1988). Testicular cancer in young Norwegians. *J. Surg. Oncol.*, **39**, 43.

FOSSA, S.D., NESLAND, J.M., WÆHRE, H., AMELLEM, Ø. & PETTERSEN, E.O. (1991). DNA ploidy in the primary tumor from patients with non-seminomatous testicular gern cell tumors clinical stage I. *Cancer* (in press).

FOSSA, S.D. & THORUD, E. (1986). DNA flow cytometry of cells obtained from old paraffin-embedded specimens. A comparison with results of scanning absorption cytometry. *Path. Res. Pract.*, **181**, 200.

FRIEDMAN, N.B. (1951). The comparative morphogenesis of extragenital and gonadal teratoid tumors. *Cancer*, **4**, 265.

GUSTAFSON, H., TRIBUKAIT, B. & ESPOSTI, P.L. (1982). DNA profile and tumour progression in patients with superficial bladder tumours. *Urol. Res.*, **10**, 13.

HEDLEY, D.W., FRIEDLANDER, M.L., TAYLOR, I.W., RUGG, C.A. & MUSGROVE, E.A. (1983). Method for analysis of cellular DNA content of paraffin-embedded pathological material using flow cytometry. *J. Histochem. Cytochem.*, **31**, 1333.

HIDDEMAN, W., SCHUMANN, J., ANDREEF, M. & 6 others (1984). Convention on nomenclature for DNA cytometry. *Cancer Genet. Cytogenet.*, **13**, 181.

JACOBSEN, A.B., THORUD, E., FOSSA, S.D. & 4 others (1988). DNA flow cytometry in malignant melanomas. A comparison of results from fresh and paraffin-embedded material. *Virchows Archiv. Cell Pathol.*, **54**, 273.

KAERN, J., TROPE, C., KJORSTAD, K.E., ABELER, V. & PETTERSEN, E.O. (1990). Cellular DNA content as a new prognostic tool in patients with borderline tumours of the ovary. *Gynecol. Oncol.*, **38**, 452.

KALLIONIEMI, P.-O., HIETANEN, T., MATTILA, J., LEHTINEN, M., LAUSLAHTIK, K. & KOIVULA, T. (1987). Aneuploid DNA content and high S-phase fraction of tumour cells are related to poor prognosis in patients with primary breast cancer. *Eur. J. Cancer Clin. Oncol.*, **23**, 277.

MARTINEAU, M. (1969). Chromosomes in human testicular tumours. *J. Pathol.*, **99**, 271.

NATIV, O., WINKLER, H.Z., FARROW, G.M. & LIEBER, M.M. (1989). Nonseminomatous germ cell tumours of the testis (NSGCTT): relation of nuclear DNA ploidy to tumour behaviour (meeting abstract). *J. Urol.*, **141**, 298A.

OLIVER, R.T.D. (1987). HLA phenotype and clinicopathological behaviour of germ cell tumours: possible evidence for clonal evolution from seminomas to nonseminomas. *Int. J. Androl.*, **10**, 85.

OOSTERHUIS, J.W., CASTEDO, S.M.M.J., DE JONG, B. & 4 others (1989). Ploidy of primary germ cell tumours of the testis. *Labor. Invest.*, **60**, 14.

O'REILLY, S.M., CAMPLEJOHN, R.S., BARNES, D.M., MILLIS, R.R., RUBENS, R.D. & RICHARDS, M.A. (1990). Node-negative breast cancer: prognostic subgroups defined by tumor size and flow cytometry. *J. Clin. Oncol.*, **8**, 2040.

PECKHAM, M.J., BARRETT, A., MCELWAIN, T.J. & HENDRY, W.F. (1979). Combined management of malignant teratoma of the testis. *Lancet*, **ii**, 267.

PIERCE, G.B. & ABELL, M.R. (1972). Embryonal carcinoma of the testis. *Pathol. Annu.*, **5**, 27.

QUIRKE, P., DYSON, J.E.D., SUTTON, J., ANDERSON, C.K., JOSLIN, C.A.F. & BIRD, C.C. (1986). Assessment of germ cell tumours of testis by flow cytometry and histopathology. *Adv. Biosci.*, **55**, 45.

RAGHAVAN, D., SULLIVAN, A.L., PECKHAM, M.J. & NEVILLE, M. (1982). Elevated serum alphaphoetoprotein and seminoma. *Cancer*, **50**, 982.

SLEDGE, G.W., EBLE, J.N., ROTH, B.J., WUHRMAN, B.P. & EINHORN, L.H. (1987). Flow cytometry derived DNA content of the primary lesions of advanced germ cell tumours. *Int. J. Androl.*, **10**, 115.

STEEN, H.B. & LINDMO, T. (1979). Flow-cytometry: a high-resolution instrument for everyone. *Science*, **204**, 403.

TRIBUKAIT, B. (1987). Flow cytometry in assessing the clinical aggressiveness of genito-urinary neoplasms. *World J. Urol.*, **5**, 108.